# Assessing the impact of proton versus photon therapy on health-related quality of life in lung cancer

**DOI:** 10.1016/j.ctro.2026.101160

**Published:** 2026-04-06

**Authors:** Bradley M. Sugden, Willem J.A. Witlox, Francesco Cortiula, Lizza E.L. Hendriks, Judith van Loon, Maria Jacobs, Djoya Hattu, Manuela A. Joore, Bram L.T. Ramaekers, Dirk K.M. De Ruysscher

**Affiliations:** a Department of Clinical Epidemiology and Medical Technology Assessment, Maastricht University Medical Centre+ (MUMC+), Maastricht, the Netherlands; b Department of Radiation Oncology (Maastro Clinic), Maastricht University Medical Centre+ (MUMC+), GROW School on Oncology and Developmental Biology, Maastricht, the Netherlands; c Tilburg School of Economics and Management, Tilburg University, Tilburg, the Netherlands

**Keywords:** Health-related quality of life, Proton, Photon, Lung cancer, Utility

## Abstract

**Background and purpose:**

A more conformal dose deposition of intensity-modulated proton therapy (IMPT) may enhance health-related quality of life (HRQoL) through reduced radiation-induced toxicities, versus photon-based intensity-modulated radiotherapy (IMRT). However, most patients are selected for IMPT based on relative reduction in mean heart dose and correlated 2-year mortality. A HRQoL difference would thus not necessarily be expected. This study evaluates HRQoL outcomes in patients with lung cancer treated with IMPT versus IMRT.

**Materials and methods:**

Patients considered for IMPT via the model-based approach and treated with IMPT or IMRT were included. HRQoL was measured using EQ-5D-5 L, EQ-VAS, and EORTC QLQ-C30 Global Health Status (GHS) at baseline, 3, 6, 12 months, and annually up to 5 years. Generalised linear mixed effects (GLM) models were developed in R to assess the impact of radiation modality on HRQoL. Missing data were handled using multiple imputation with propensity score weighting to balance treatment groups. Radiation modality, WHO performance status, baseline HRQoL, and baseline dyspnoea were included as fixed effects in final models. Minimal important difference cut-offs were 0.03 (EQ-5D-5 L), and 0.05 (EQ-VAS; GHS).

**Results:**

171 IMPT and 71 IMRT patients were included (mean follow-up: 8 months). Disease was predominantly stage III non-small cell lung cancer and most patients received chemotherapy. Baseline mean HRQoL scores were similar between groups. Coefficient estimates for IMPT versus IMRT were −0.022 (EQ-5D-5 L), −0.008 (EQ-VAS) and −0.014 (GHS). Differences were not deemed statistically significant nor clinically relevant.

**Conclusion:**

Certain clinical advantages for IMPT have previously been demonstrated, but no significant difference in HRQoL between IMPT and IMRT was observed.

## Highlights

• Current model-based selection may not lead to observable HRQoL benefits.
• (Cost-)effectiveness analyses should not assume utility gains from IMPT alone.
• Methodological rigour through multiple imputation and propensity score weighting.
• Open access to full analyses coding.
• (Cost-)effectiveness analyses should focus on IMPT toxicity reduction and survival.

## Introduction

Radiotherapy represents an important treatment modality for patients with lung cancer, which has previously been shown to improve loco-regional recurrence and survival [1]. Previous research found that Intensity-modulated proton radiotherapy (IMPT) has shown benefits over photon-based intensity-modulated radiotherapy (IMRT), including lower rates of severe lymphopenia and anaemia, improved performance status following concurrent chemoradiotherapy (CCRT), and increased likelihood of receiving adjuvant durvalumab [2]. Health-related quality of life (HRQoL) represents an individual’s overall wellbeing as it is affected by their health state [3]. IMPT may enhance HRQoL by reducing radiation-induced toxicities through lowering the radiation exposure to neighbouring organs at risk through a highly conformal dose deposition at the target, relative to IMRT [4], [5], [6]. The severity of treatment-induced toxicities is dependent upon many factors such as individual patient heterogeneity in the radiosensitivity of normal tissues, and the volume of irradiated normal tissues. Radiation-induced toxicity can be either acute or late, both of which can affect short- and long-term patient-reported health-related quality of life (HRQoL) and, in extreme cases, lead to death.

For lung cancer in The Netherlands, IMPT eligibility is determined in terms of reduced probability of radiation-induced toxicities using a model-based approach. The model-based approach is based on the observation that the probability of radiation-induced toxicities depends on the volumes of organs at risk that receive certain doses of radiation [7]. Currently, patients with lung cancer are selected based on normal tissue complication probability (NTCP) models for: mortality [8], oesophagitis [9], and pneumonitis [10].

The impact of IMRT on HRQoL in patients with lung cancer has previously been studied in The Netherlands [11], [12], [13], [14], [15], [16], [17]. Baseline HRQoL varied across study populations, however all studies observed either stable HRQoL scores overtime or a short-term but reversible decrease. A negative impact of radiation-induced toxicity on HRQoL has also been demonstrated in breast cancer and head and neck cancer [18], [19], [20]. Further, as part of the multicentre REQUITE study, the adverse impact of clinician-scored symptoms/toxicity on HRQoL was demonstrated in patients with early-stage and locally-advanced NSCLC, showing variation in the impact of acute and late toxicities on HRQoL. Toxicities during CCRT and 3-dimensional radiotherapy had the largest impact on HRQoL [21].

While the more conformal dose deposition of IMPT relative to IMRT may lead to a consequential HRQoL benefit through a reduced lung dose [2], [22], [23], the expected difference for patients selected using the model-based approach remains unclear. In practice, most patients are selected for protons based on the relative reduction in mean heart dose and correlated 2-year mortality [24], [8]. As such, a difference in HRQoL would thus not necessarily be expected. Furthermore, the average dose to the oesophagus is higher with IMPT than for IMRT, which can lead to increased pain when swallowing, with associated negative implications on HRQoL being previously demonstrated [17].

To the best of our knowledge, the comparative impact of IMPT (versus IMRT) on HRQoL has not been previously studied in patients with lung cancer.

The present study aims to assess the impact of IMPT versus IMRT on HRQoL for patients with lung cancer in The Netherlands from a single combined IMPT and IMRT centre selected using the model-based approach.

## Methods

### Materials

#### Available data

To assess the impact of radiation modality (IMPT vs IMRT) on HRQoL, an observational dataset was used, comprising consecutive patients with lung cancer in a combined proton and photon radiotherapy centre in The Netherlands. Patients with lung cancer referred for conventionally fractionated radiotherapy with curative intent that received an IMPT treatment planning comparison were included, irrespective of tumour stage or histology. Patients that received IMRT without a treatment planning comparison were excluded from the analysis. This approach ensures patients with the same indication and referral pathway and thus, in principle, were eligible for either treatment modality. This avoids comparison with IMRT patients that may differ systematically and hence, were not considered for a treatment planning comparison. Treatments were administered between October 2019 and November 2023. Included patients were treated with IMPT, IMRT (or a combination thereof) alone or combined with surgery and/or chemotherapy. Patients that received at least 30% of total fractions as protons were classified as IMPT for the analysis, consistent with the approach utilised by Cortiula et al. [2]. However, given the uncertainty for this cut-off in the context of HRQoL, alternative cut-offs were explored in a scenario analysis. Patients treated with IMRT that did not receive a planning comparison were not included.

Patient-reported outcome measures (PROMs) routinely captured for patients treated with proton or photon therapy for lung cancer were utilised to assess the impact of each modality on patient HRQoL. PROMs were issued before first treatment, during radiotherapy (month one), three months, six months, one year, and each year thereafter up to five years. Generic HRQoL was measured using the EuroQol-5D questionnaire (five level version, EQ-5D-5 L). Responses were translated to utility scores using the Dutch tariff [25]. HRQoL was also measured using the EQ-VAS, a visual analogue scale from 0 (worst possible health imaginable) to 100 (best possible health imaginable). Disease-specific HRQoL was assessed using the EORTC QLQ-C30 instrument. Specifically used in the present analyses, the instruments global health status (GHS) scale linearly converts two items for overall health and quality of life to a 0–100 point scale with higher scores representing higher levels of HRQoL. EQ-VAS and GHS scores were divided by 100 to derive utility scores between 0 and 1.

This study was approved by the institutional research committee (METC number 2023-0048).

### Statistical analysis

Baseline patient characteristics, PROM questionnaire compliance, and HRQoL scores over time were analysed using descriptive statistics. To negate a potential reduction in statistical power due to missing data, we imputed missing data under the assumption that this was missing at random. The model-based approach to determining patient eligibility for IMPT introduces potential selection bias due to the inclusion of patient characteristics in NTCP models. As such, two methods were explored to balance baseline differences between patients in the IMPT and IMRT groups. To estimate the treatment effect overtime and to account for non-independent repeated measures of PROMs within patients, in addition to handling the potential for confounding, mixed effects models were utilised for the analysis. Such statistical methods are explained in more detail below.

Missing data imputation was conducted using multiple imputation by chained equations (*mice* package). Due to potential selection bias resulting from IMPT eligibility being determined through a model-based approach, we conducted propensity score weighting to balance treatment group baseline characteristics (base-case). Given a lack of consensus on the best approach to balance, genetic matching was explored in a scenario analysis following the application by Pouwels *et al*. [26], as was results with no matching/weighting. For propensity score weighting, firstly baseline characteristics were analysed using density plots to ensure sufficient overlap between treatment groups. Propensity score weighting was applied using the *Toolkit for Weighting and Analysis of Nonequivalent Groups* (twang) package in R [27]. This package utilises gradient boosted models to estimate propensity scores. Convergence was assessed using the plot() function in R. Covariate balance before and after weighting was assessed using standardised mean differences, variance ratios between treatment groups, Kolmogorov-Smirnov (KS) p-values for continuous variables, and T-test p-values. Relative to the explored genetic matching scenario, propensity score weighting has the advantage of keeping all observations for subsequent analyses. Disease stage, age at start of radiotherapy, WHO performance status (WHO-PS), baseline EQ-5D-5 L utility, and baseline dyspnoea were included in the final weighting.

Generalised linear mixed effects (GLM) models with random intercepts (with Gaussian distribution and identity link) were developed in R (lme4 package) to estimate the impact of protons vs photons on HRQoL metrics. Separate models were explored with EQ-5D-5 L index scores, EQ-5D VAS scores, and GHS scores as the dependent variables. Patient identifiers were included as a random effects variable to model variability due to grouping structures. Expert consultation was used to identify clinically plausible fixed effects variables as a starting point for inclusion. Age (start of radiotherapy), gender, WHO-PS, baseline HRQoL, stage, daily fractionation frequency, pulmonary comorbidity, durvalumab status (note: durvalumab treatment starts ≤ 3 months post-RT), chemotherapy sequence, smoking status, surgery status, treatment adaptation, tumour location, histology, total radiation dose received, baseline dyspnoea, baseline dysphagia, and gross tumour volume (GTV) were all considered as candidate variables for inclusion. Multicollinearity of candidate fixed effects variables was assessed by computing generalised variance inflation factors (car package). Stepwise model selection was utilised through computing a Wald statistic for model iterations whereby one fixed effects variable was removed at a time. The Wald test method was used (*mice* package in R [28]) to determine variable inclusion in the final models (α = 0.05). Here, pooled results of the separately analysed multiply imputed datasets were utilised. Treatment allocation was forced into the model during selection. Minimal important difference cut-offs, utilised to determine clinical relevance, were 0.03 for EQ-5D-5 L and 0.05 points for EQ-VAS and GHS HRQoL measurements [29], [30]. The selection process was conducted on models with EQ-5D-5 L as the outcome variable. For comparability, models for EQ-5D VAS and GHS were fitted with the same covariates.

To assess the impact of treatment on HRQoL, the base-case analysis was conducted in conjunction with scenario analyses to show the relative impact of certain assumptions. The conducted scenarios were: 1) genetic matching (propensity score weighting in base-case); 2) impact of defining the proton group as those that received at least 50% of total fractions as protons (30% in base-case); 3) impact of defining the proton group as those that received at least 80% of total fractions as protons (30% in base-case); 4) impact of defining the proton group as those that received at least 100% of total fractions as protons (30% in base-case); 5) no propensity score weighting or genetic matching approach to balance covariates (propensity score weighting used in base-case).

Full code for the present analyses is openly available here: https://github.com/bmsugden/HRQoL_Lung_Proton

## Results

In total, 242 patients were included in the analysis, 171 (71%) of which were classified as IMPT and 71 (29%) as IMRT. Of the IMPT group, 39 (23%) received protons only, whereas the remaining 132 (77%) receiving a combination of protons and photons. Also, 66 (93%) of the IMRT group received photons only with the remaining 5 (7%) receiving a combination. Baseline characteristics were similar, with some notable differences. A higher proportion of patients treated with IMPT (92%) had a NSCLC tumour histology, compared with IMRT (83%), with a higher proportion of stage I-II (16% for IMPT versus 4% for IMRT). All baseline patient and treatment characteristics, and baseline HRQoL scores, are presented in Table 1. Raw baseline patient and treatment characteristics (pre-imputation) and missing values are presented in Appendix A.

**Table 1:** Baseline patient and treatment characteristics before and after propensity score weighting (post imputation – aggregated across imputed datasets).

Variable		Protons (SD/%)	Photons (SD/%)	Total (SD/%)
Number of patients		171	71	242
**Patient characteristics**				
Mean age		67.57 (9.32)	67.03 (8.29)	67.41 (9.02)
Sex (female)		77 (45%)	36 (51%)	113 (47%)
Smoking status				
	Current	37 (22%)	16 (23%)	53 (22%)
	Former	126 (73%)	50 (70%)	176 (73%)
	Never	8 (5%)	5 (7%)	13 (5%)
WHO PS				
	0	31 (18%)	17 (24%)	48 (20%)
	1	110 (64%)	37 (52%)	147 (61%)
	2 or higher	30 (18%)	17 (24%)	47 (19%)
Histology				
	NSCLC	158 (92%)	59 (83%)	217 (89%)
	SCLC	13 (8%)	12 (17%)	25 (11%)
Stage				
	I/II	26 (15%)	3 (4%)	29 (12%)
	III	127 (74%)	57 (80%)	184 (76%)
	IV	17 (10%)	11 (16%)	28 (12%)
Tumour location				
	Left/both lungs	77 (45%)	48 (68%)	125 (52%)
	Other	94 (55%)	23 (32%)	117 (48%)
Pulmonary comorbidity (yes)		82 (48%)	36 (51%)	118 (49%)
Gross Tumour Volume		88.89 (104.84)	86.39 (112.32)	88.16 (106.93)
Baseline dyspnoea (grade 1 or 2)		99 (58%)	38 (54%)	137 (57%)
Baseline dysphagia (grade 1 or 2)		10 (6%)	16 (23%)	27 (11%)
**Baseline health-related quality of life**				
EQ-5D-5 L		0.80 (0.16)	0.81 (0.14)	0.80 (0.16)
EQ-VAS		0.68 (0.18)	0.73 (0.18)	0.70 (0.17)
EORTC QLQ-C30 GHS		0.71 (0.19)	0.72 (0.18)	0.72 (0.19)
**Treatment characteristics**				
Received combination of proton and photon				
	Yes	132 (77%)	5 (7%)	137 (57%)
	No	39 (23%)	66 (93%)	105 (43%)
Chemotherapy sequence				
	Concurrent	104 (61%)	44 (62%)	148 (61%)
	Sequential	40 (23%)	22 (31%)	62 (26%)
	None	27 (16%)	5 (7%)	32 (13%)
Surgery (yes)		8 (5%)	3 (4%)	11 (5%)
Durvalumab (yes)*		79 (51%)	28 (44%)	107 (49%)
Mean radiation dose received (Gy)		58.43 (5.33)	57.34 (5.96)	58.11 (5.53)
Fractionation daily frequency				
	Once daily	157 (92%)	60 (85%)	217 (90%)
	Twice daily	14 (8%)	11 (16%)	25 (10%)

Percentages may not total 100% due to rounding.

*Percentage of patients with stage III NSCLC that received durvalumab.

Abbreviations: GHS = (EORTC QLQ-C30) global health status, NSCLC = non-small cell lung cancer, SCLC = small cell lung cancer, SD = standard deviation, WHO-PS = World Health Organisation Performance Status.

Balance assessments for covariates included for propensity score weighting and genetic matching are presented in Appendix B.

**Fig. 1:**
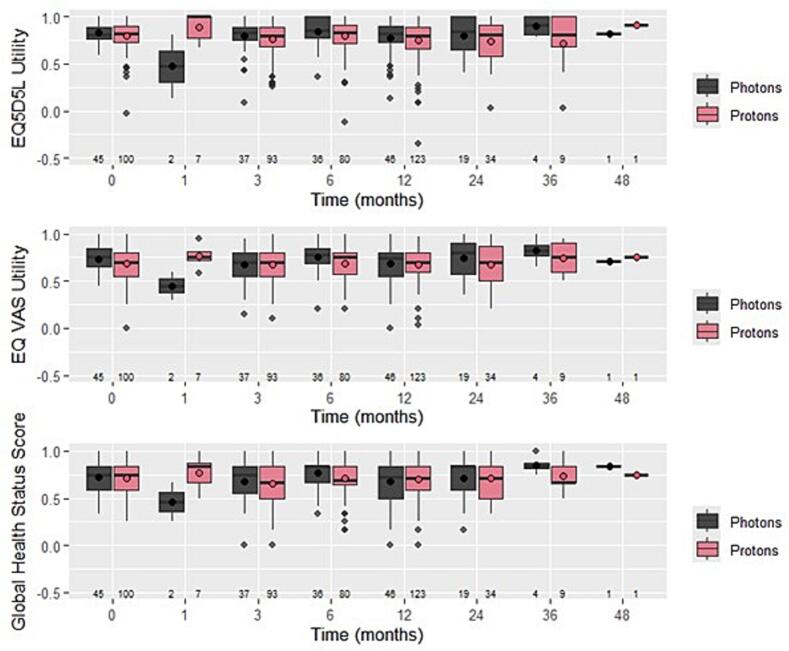
Boxplots visualising EQ-5D-5 L utility scores, EQ VAS scores, and EORTC QLQ-C30 global health status scores between IMPT and IMRT. Open dots represent averages over time. Abbreviations: VAS = visual analogue scale.

Fig. 1 shows boxplots visualising HRQoL scores over time between protons and photons. The three measurements of HRQoL show similar patterns. These were stable over time except for month one (i.e., during radiotherapy).

The final base-case models included treatment allocation (IMPT or IMRT), baseline HRQoL, WHO-PS, and presence of baseline (grade 1 or 2) dyspnoea as fixed effects variables. The impact of protons versus photons was not deemed to be clinically relevant nor statistically significant in the final EQ-5D-5 L model (coefficient estimate: −0.022; p-value: 0.366), the final EQ-5D VAS model (coefficient estimate: −0.008; p-value: 0.710), nor the final global health status model (coefficient estimate: −0.014; p-value: 0.552).

Full base-case results are presented in Table 2.

**Table 2:** Base case analyses results.

HRQoL instrument	HRQoL outcome measure	Coefficient			SE of pooled estimates	*p*-value	95% confidence interval
***Base case analyses*** ***(analysis of imputed data with propensity score weighting)***							
EQ-5D-5 L	Utility score	Intercept*		0.475	0.086	0.000	0.305, 0.645
		*Treatment allocation*					
			IMPT	−0.022	0.025	0.366	−0.071, 0.026
		*WHO-PS*					
			1	−0.054	0.026	0.039	−0.105, −0.003
			2+	−0.086	0.036	0.018	−0.156, −0.015
		*Baseline HRQoL*		0.494	0.089	0.000	0.318, 0.669
		*Baseline dyspnoea (grade 1 or 2)*		−0.056	0.026	0.034	−0.108, −0.004
	VAS	Intercept*		0.504	0.066	0.000	0.374, 0.634
		*Treatment allocation*					
			IMPT	−0.008	0.023	0.710	−0.053, 0.036
		*WHO-PS*					
			1	−0.066	0.027	0.015	−0.119, −0.013
			2+	−0.104	0.037	0.005	−0.177, −0.031
		*Baseline HRQoL*		0.384	0.072	0.000	0.243, 0.525
		*Baseline dyspnoea (grade 1 or 2)*		−0.043	0.027	0.107	−0.095, 0.009
EORTC QLQ-C30	Global Health Status	Intercept*		0.507	0.060	0.000	0.389, 0.625
		*Treatment allocation*					
			IMPT	−0.014	0.023	0.552	−0.059, 0.032
		*WHO-PS*					
			1	−0.072	0.028	0.011	−0.128, −0.016
			2+	−0.125	0.039	0.001	−0.201, −0.049
		*Baseline HRQoL*		0.388	0.065	0.000	0.260, 0.515
		*Baseline dyspnoea (grade 1 or 2)*		−0.032	0.029	0.271	−0.090, 0.025

EQ-VAS and EORTC QLQ-C30 scores were divided by 100 prior to analysis for comparability (minimal important difference is therefore 0.05).

*Reference values were as follows: Treatment allocation = IMRT, WHO-PS = 0, Baseline HRQoL = 0.

Abbreviations: HRQoL = health-related quality of life, SE = standard error, VAS = visual analogue scale, WHO-PS = World Health Organisation Performance Status.

Full results of the scenario analyses are presented in Table 3. Baseline WHO-PS was not included in the final models as a fixed effect variable in the scenario analysis (1) using genetic matching to balance treatment group baseline characteristics. Fixed effects in the final models for the scenario analysis (5) with no propensity score weighting or genetic matching were the same as the base-case. The results of both scenarios were similar to the base-case results.

**Table 3:** Scenario analyses results.

HRQoL instrument	HRQoL outcome measure	Coefficient			SE of pooled estimates	*p*-value	95% confidence interval
***Scenario analyses 1*** ***(analysis of imputed data with genetic matching)***							
EQ-5D-5 L	Utility score	Intercept*		0.434	0.086	0.000	0.264, 0.605
		*Treatment allocation*					
			IMPT	−0.029	0.028	0.302	−0.083, 0.026
		*Baseline HRQoL*		0.510	0.091	0.000	0.332, 0.689
		*Baseline dyspnoea (grade 1 or 2)*		−0.083	0.024	0.001	−0.130, −0.036
	VAS	Intercept*		0.443	0.068	0.000	0.309, 0.576
		*Treatment allocation*					
			IMPT	−0.015	0.026	0.553	−0.066, 0.035
		*Baseline HRQoL*		0.420	0.076	0.000	0.270, 0.570
		*Baseline dyspnoea (grade 1 or 2)*		−0.071	0.026	0.006	−0.122, −0.020
EORTC QLQ-C30	Global Health Status	Intercept*		0.450	0.061	0.000	0.329, 0.570
		*Treatment allocation*					
			IMPT	−0.022	0.028	0.420	−0.077, 0.032
		*Baseline HRQoL*		0.414	0.070	0.000	0.276, 0.551
		*Baseline dyspnoea (grade 1 or 2)*		−0.069	0.026	0.008	−0.120, −0.018

***Scenario Analyses 2*** ***(Analysis of imputed data with propensity score weighting; proton patients receive ≤ 50% fractions as protons. Base case = 30%)***							
EQ-5D-5 L	Utility Score	Intercept*		0.478	0.086	0.000	0.309, 0.647
		*Treatment allocation*					
			IMPT	−0.027	0.024	0.266	−0.074, 0.021
		*WHO-PS*					
			1	−0.054	0.026	0.037	−0.106, −0.003
			2+	−0.089	0.036	0.014	−0.159, −0.018
		*Baseline HRQoL*		0.494	0.089	0.000	0.318, 0.669
		*Baseline dyspnoea (grade 1 or 2)*		−0.055	0.026	0.036	−0.107, −0.004
	VAS	Intercept*		0.504	0.065	0.000	0.377, 0.632
		*Treatment allocation*					
			IMPT	−0.010	0.022	0.647	−0.053, 0.033
		*WHO-PS*					
			1	−0.066	0.027	0.014	−0.119, −0.013
			2+	−0.105	0.037	0.005	−0.178, −0.032
		*Baseline HRQoL*		0.385	0.071	0.000	0.244, 0.525
		*Baseline dyspnoea (grade 1 or 2)*		−0.043	0.027	0.110	−0.095, 0.010
EORTC QLQ-C30	Global Health Status	Intercept*		0.505	0.059	0.000	0.388, 0.621
		*Treatment allocation*					
			IMPT	−0.011	0.022	0.615	−0.055, 0.033
		*WHO-PS*					
			1	−0.073	0.028	0.011	−0.129, −0.017
			2+	−0.126	0.039	0.001	−0.202, −0.050
		*Baseline HRQoL*		0.388	0.065	0.000	0.261, 0.516
		*Baseline dyspnoea (grade 1 or 2)*		−0.032	0.029	0.278	−0.089, 0.026

***Scenario Analyses 3*** ***(Analysis of imputed data with propensity score weighting; proton patients receive ≤ 80% fractions as protons. Base case = 30%)***							
EQ-5D-5 L	Utility Score	Intercept*		0.453	0.082	0.000	0.290, 0.615
		*Treatment allocation*					
			IMPT	0.007	0.021	0.733	−0.034, 0.048
		*WHO-PS*					
			1	−0.057	0.026	0.031	−0.109, −0.005
			2+	−0.086	0.036	0.018	−0.156, −0.015
		*Baseline HRQoL*		0.499	0.089	0.000	0.324, 0.674
		*Baseline dyspnoea (grade 1 or 2)*		−0.054	0.026	0.043	−0.106, −0.002
	VAS	Intercept*		0.491	0.062	0.000	0.369, 0.613
		*Treatment allocation*					
			IMPT	0.007	0.019	0.712	−0.031, 0.045
		*WHO-PS*					
			1	−0.067	0.027	0.013	−0.120, −0.014
			2+	−0.104	0.037	0.006	−0.177, −0.031
		*Baseline HRQoL*		0.388	0.071	0.000	0.249, 0.527
		*Baseline dyspnoea (grade 1 or 2)*		−0.041	0.026	0.118	−0.093, 0.011
EORTC QLQ-C30	Global Health Status	Intercept*		0.492	0.058	0.000	0.378, 0.605
		*Treatment allocation*					
			IMPT	0.014	0.021	0.489	−0.027, 0.055
		*WHO-PS*					
			1	−0.075	0.029	0.009	−0.131, −0.019
			2+	−0.125	0.039	0.001	−0.202, −0.049
		*Baseline HRQoL*		0.387	0.064	0.000	0.260, 0.514
		*Baseline dyspnoea (grade 1 or 2)*		−0.031	0.030	0.307	−0.090, 0.029

***Scenario Analyses 4*** ***(Analysis of imputed data with propensity score weighting; proton patients receive ≤ 100% fractions as protons. Base case = 30%)***							
EQ-5D-5 L	Utility Score	Intercept*		0.451	0.081	0.000	0.292, 0.611
		*Treatment allocation*					
			IMPT	0.024	0.028	0.385	−0.030, 0.078
		*WHO-PS*					
			1	−0.057	0.026	0.029	−0.109, −0.006
			2+	−0.086	0.036	0.017	−0.157, −0.015
		*Baseline HRQoL*		0.499	0.088	0.000	0.325, 0.674
		*Baseline dyspnoea (grade 1 or 2)*		−0.052	0.026	0.047	−0.103, −0.001
	VAS	Intercept*		0.489	0.061	0.000	0.370, 0.608
		*Treatment allocation*					
			IMPT	0.028	0.027	0.295	−0.024, 0.080
		*WHO-PS*					
			1	−0.068	0.027	0.012	−0.120, −0.015
			2+	−0.104	0.037	0.006	−0.177, −0.031
		*Baseline HRQoL*		0.389	0.070	0.000	0.251, 0.527
		*Baseline dyspnoea (grade 1 or 2)*		−0.039	0.026	0.129	−0.090, 0.012
EORTC QLQ-C30	Global Health Status	Intercept*		0.493	0.057	0.000	0.380, 0.606
		*Treatment allocation*					
			IMPT	0.034	0.029	0.241	−0.023, 0.090
		*WHO-PS*					
			1	−0.075	0.028	0.009	−0.131, −0.019
			2+	−0.125	0.039	0.001	−0.202, −0.049
		*Baseline HRQoL*		0.387	0.065	0.000	0.260, 0.514
		*Baseline dyspnoea (grade 1 or 2)*		−0.029	0.030	0.326	−0.088, 0.029

***Scenario analysis 5*** ***(analysis of imputed data – no propensity score weighting or matching)***							
EQ-5D-5L	Utility score	Intercept*		0.481	0.086	0.000	0.312, 0.650
		*Treatment allocation*					
			IMPT	−0.023	0.027	0.388	−0.076, 0.029
		*WHO-PS*					
			1	−0.056	0.027	0.037	−0.108, −0.003
			2+	−0.087	0.036	0.018	−0.159, −0.015
		*Baseline HRQoL*		0.492	0.088	0.000	0.319, 0.664
		*Baseline dyspnoea (grade 1 or 2)*		−0.061	0.026	0.020	−0.113, −0.010
	VAS	Intercept*		0.508	0.066	0.000	0.379, 0.638
		*Treatment allocation*					
			IMPT	−0.010	0.024	0.687	−0.057, 0.037
		*WHO-PS*					
			1	−0.066	0.027	0.014	−0.118, –0.014
			2+	−0.101	0.036	0.006	−0.172, −0.029
		*Baseline HRQoL*		0.380	0.070	0.000	0.242, 0.519
		*Baseline dyspnoea (grade 1 or 2)*		−0.046	0.026	0.077	−0.097, 0.005
EORTC QLQ-C30	Global Health Status	Intercept*		0.511	0.060	0.000	0.394, 0.629
		*Treatment allocation*					
			IMPT	−0.016	0.024	0.504	−0.063, 0.031
		*WHO-PS*					
			1	−0.071	0.028	0.011	−0.127, −0.016
			2+	−0.123	0.038	0.001	−0.198, −0.049
		*Baseline HRQoL*		0.385	0.063	0.000	0.260, 0.510
		*Baseline dyspnoea (grade 1 or 2)*		−0.036	0.028	0.201	−0.090, 0.019

EQ-VAS and EORTC QLQ-C30 scores were divided by 100 prior to analysis for comparability (minimal important difference is therefore 0.05).

*Reference values were as follows: Treatment allocation = IMRT, WHO-PS = 0, Baseline HRQoL = 0.

Abbreviations: HRQoL = health-related quality of life, SE = standard error, VAS = visual analogue scale, WHO-PS = World Health Organisation Performance Status.

For scenarios utilising a different cut-offs (50%, 80%, 100%) to determine presence in the IMPT group (30% in base-case), the impact of IMPT versus IMRT in all final models was not deemed to be clinically relevant nor statistically significant. However, there was a relative improvement in the regression coefficients of IMPT compared with the base-case, suggesting that the impact of receiving a combination of IMPT and IMRT had a negative impact on HRQoL, compared with both protons-only and photons-only.

## Discussion

This study assessed the impact of IMPT, compared with IMRT, on generic and disease-specific HRQoL in patients with lung cancer, that were considered for IMPT (i.e., received a treatment planning comparison).

No clinically meaningful nor statistically significant differences in HRQoL were found for treatment with IMPT as compared with IMRT for any of the HRQoL instruments. This finding was consistent across all scenario analyses. As per the model-based approach in The Netherlands, patient eligibility for IMPT is determined based on relative capacity to benefit (versus IMRT) in terms of reduced normal tissue complication probability. That is, selection is not directly linked to expected gains in HRQoL, particularly provided that, in practice, most patients with lung cancer are selected for IMPT using the mortality model [24]. As such, results here are not contrary to expectation.

To our knowledge, this is the first study assessing the impact of IMPT versus IMRT in patients with lung cancer. Nonetheless, previous studies have assessed HRQoL within lung cancer. Grutters *et al*. [12] found EQ-5D-3 L mean utility to be 0.74 in patients surviving NSCLC (mean survival: 31.2 months), similar to the EQ-5D-5 L mean utilities for 24 months and 36 months (0.79 and 0.77, respectively) in the present study. Mean baseline EQ-5D-5 L utility in the present study (0.81) was also similar to those reported in stage III NSCLC (0.79–0.80 [15]). Mean baseline EORTC QLQ-C30 GHS was higher in the present study (0.713) than previously reported for patients with lung cancer treated with curative intent (0.527 [16]), patients with lung cancer receiving high-dose radiotherapy or CCRT (0.594 [15]), patients with inoperable stage I NSCLC (0.607 [11]; 62 [13]) and stage III NSCLC (0.629–0.650 [15]). Mean baseline EQ-VAS scores were also higher in the present study (0.696) than previously reported in stage III NSCLC (0.632–0.650 [15]). Whilst this finding may reflect poor PROMs compliance in the present study and/or reflect population differences, a similar trend was found overtime with stable HRQoL scores up to 5 years (exception of month one, which is not routinely captured and thus contains very few observations).

One potential limitation of the analyses pertains to the completion of routinely captured PROMs. Whilst an accurate assessment of expected vs actual number of PROMs observations for each follow-up moment was not possible (latest survival follow-up for clinical data was December 2022, compared with December 2023 PROMs data collection time point), PROMs compliance appeared to be low. Indeed, of the 242 patients included, only 145 (60%) patients had baseline HRQoL data. Missing data imputation was utilised to address incomplete PROMs questionnaires, however no imputation was included at follow-up moments where no questionnaire data was available. As such, the impact of compliance on model results is unclear, particularly if this restricts the measurements ability to sufficiently capture the impact of symptoms on HRQoL over time. A previous study in stage III NSCLC found PROMs questionnaire compliance was generally lower for time points later than baseline [15]. If patients with lower HRQoL are less inclined to complete PROMs questionnaires, this could potentially bias results. A need for further validation regarding the appropriateness of the presently utilised HRQoL measurements has also been highlighted in the context of advanced cancer [31]. Another potential limitation is the existence of patients receiving a combination of protons and photons as part of their treatment. That is, this limits the direct comparison of IMPT versus IMRT. We explored different cut-offs to determine whether a patient was handled as having received IMPT or IMRT (≥30% of fractions received as protons in base-case; ≥ 50%, ≥ 80%, and ≥100% in scenario analyses). Here, we found that combination treatments (i.e., fractions received with protons and photons) had a negative impact on HRQoL, relative to protons-only and photons-only (although difference remained statistically insignificant). Further, we explored the existence of a treatment plan adaptation as a fixed effect, however this was not deemed to be a significant predictor of HRQoL. Nonetheless, the IMPT group contained a relatively high proportion (77%) of patients receiving a combination of proton and photon fractions, versus IMRT (7%).

Provided patients are not assigned to IMPT or IMRT at random due to NTCP model-based selection, potential selection bias is introduced into our analyses. To account for this, we explored both propensity score weighting (base-case) and genetic matching (scenario analysis 1) to balance covariates across the treatment groups. Despite this, Soni *et al.* [32] found that propensity weighting and well-matched study populations were unable to improve agreement between observational study and randomised trial hazard ratios within oncology. This may suggest a residual selection bias remains in the context of HRQoL, despite attempts to correct for this.

One key avenue for future research would be to assess the impact of individual toxicities on HRQoL for patients with lung cancer, particularly provided that baseline dyspnoea was a significant predictor of HRQoL in the present study. Such an assessment should include toxicities within the proton indication protocol for lung cancer and those that are outside. Additionally, an assessment of the respective prevalence of each toxicity for IMPT and IMRT over time would provide greater context to the present results. Further research is required to better understand approaches to be taken in practice for improving PROMs questionnaire compliance and to address missing data. Additionally, future research should explore the impact of mixed-fraction treatment schedules on HRQoL, and potential between-centre differences.

As per findings of the present study, current NTCP-based selection may not lead to observable HRQoL benefits. Therefore, cost-effectiveness analyses should not assume utility gains from IMPT alone, and should instead focus on potential toxicity reduction versus IMRT as well as survival.

In conclusion, no meaningful difference in HRQoL was observed between IMPT and IMRT across measurements up to five years. These measurements were stable overtime and provide a good overview of the perceived health status, but they are not sufficient on their own to decide whether a treatment should be chosen without additional clinical data such as toxicity, survival, and tumour control. The results will serve to inform utility parameters for a future cost-effectiveness analysis of IMPT vs IMRT which will further need to account for differences between IMPT and IMRT in terms of disease progression, survival, subsequent treatments, toxicities, and associated costs.

## Declaration of competing Interest

The authors declare the following financial interests/personal relationships which may be considered as potential competing interests: **Sugden BM**, **Witlox WJA**, **Jacobs M**, **Hattu J**, **Joore M**, and **Ramaekers BLT** declare that they have no known competing financial interests or personal relationships that could have appeared to influence the work reported in this paper; **Cortiula F:** Speaker educationals/webinars: AstraZeneca Roche, Johnson and Johnson (self). Regeneron, MSD (self). Local PI of clinical trial: AstraZeneca, MSD (All payments to institution). Non-financial: Member guideline committees: ESCO guidelines on metastatic NSCLC (non-financial). **Hendriks LEL:** Research funding: Roche Genentech, AstraZeneca, Boehringer Ingelheim, Takeda, Merck, Pfizer, Novartis, Gilead. Summit under negotiation, Amgen under negotiation (All payments were paid to the institution). Speaker educationals/webinars: AstraZeneca, Bayer, Lilly, MSD, high5oncology, Takeda, Janssen, GSK, Sanofi, Pfizer (Inst), Medtalks, Benecke, VJOncology, Medimix (self) (All payments paid to the institution with the exception of Medtalks, Benecke, VJOncology, Medimix). Advisory boards: Abbvie, Amgen, Anhearth, AstraZeneca, Bayer, BMS, Boehringer Ingelheim, Daiichi, GSK, Gilead, Janssen, Lilly, Merck, MSD, Novartis, Pfizer, Pierre Fabre, Roche, Sanofi, Summit Therapeutics, Takeda (All payments were paid to the institution). Member guideline committees: Dutch guidelines on NSCLC, brain metastases and leptomeningeal metastases (payment to self), ESMO guidelines on metastatic NSCLC and SCLC (non-financial), Other (non-financial): former secretary and current chair NVALT studies foundation, former subchair EORTC metastatic NSCLC systemic therapy and current secretary of this group, vice-chair scientific committee Dutch Thoracic Group. Local PI of clinical trials: AstraZeneca, GSK, Novartis, Merck, Roche, Takeda, Blueprint, Mirati, Abbvie, Gilead, MSD, Merck, Amgen, Boehringer Ingelheim, Pfizer, Daiichi, Amgen, BMS (All payments to institution). **De Ruysscher D:** Support for present manuscript: BMS (No personal financial interests). Research grant/support/Advisory Board: AstraZeneca, BMS, Beigene, Philips, Olink (Instititutional financial interests – no personal financial interests). Advisory Board: Eli-Lilly (institutional financial interests – no personal financial interests).
