# Supplementary material for: Assessing the impact of proton versus photon therapy on health-related quality of life in lung cancer
Source: Clin Transl Radiat Oncol. 2026 Apr 6;59:101160. doi: 10.1016/j.ctro.2026.101160 (PMC13101553; doi:10.1016/j.ctro.2026.101160)
Supplement: Supplementary Data 1 [file mmc1.docx]

**Supplementary Appendices**

**Appendix A: Baseline characteristics and missing data overview (pre-imputation)**

| **Variable** | | | **Protons** | **Photons** | **Total**  **(SD/% of valid)** | **Missing** |
| --- | --- | --- | --- | --- | --- | --- |
| Number of patients | | | 171 (100%) | 71 (100%) | 242 (100%) | 0 (0%) |
| **Baseline Characteristics** | | | | | | |
| Mean age | | | 67.6 (9.3) | 67.0 (8.3) | 67.4 (9.0) | 0 (0%) |
| Sex (female) | | | 77 (45%) | 36 (51%) | 113 (47%) | 0 (0%) |
| Smoking status | | |  |  |  | 0 (0%) |
|  | | Current | 37 (22%) | 16 (23%) | 53 (22%) |  |
|  | | Former | 126 (74%) | 50 (70%) | 176 (73%) |  |
|  | | Never | 8 (5%) | 5 (7%) | 13 (5%) |  |
| WHO PS | | |  |  |  | 0 (0%) |
|  | | 0 | 31 (18%) | 17 (24%) | 48 (20%) |  |
|  | | 1 | 110 (64%) | 37 (52%) | 147 (61%) |  |
|  | | 2 | 30 (18%) | 17 (24%) | 47 (19%) |  |
| Histology | | |  |  |  | 0 (0%) |
|  | | NSCLC | 146 (92%) | 57 (83%) | 203 (89%) |  |
|  | | SCLC | 13 (8%) | 12 (17%) | 25 (11%) |  |
| Stage | | |  |  |  | 0 (0%) |
|  | | I/II | 26 (15%) | 3 (4%) | 29 (12%) |  |
|  | | III | 127 (75%) | 56 (80%) | 183 (76%) |  |
|  | | IV | 17 (10%) | 11 (16%) | 28 (12%) |  |
| Tumour location | | |  |  |  | 19 (8%) |
|  | | Left/both lungs | 71 (46%) | 45 (67%) | 116 (52%) |  |
|  | | Other | 85 (55%) | 22 (33%) | 107 (48%) |  |
| Pulmonary comorbidity (yes) | | | 82 (48%) | 36 (51%) | 118 (49%) | 0 (0%) |
| Gross Tumour Volume | | | 85.2 (99.2) | 85.4 (111.2) | 85.2 (102.6) | 13 (5%) |
| Baseline dyspnoea (grade 1 or 2) | | | 89 (58%) | 3 (60%) | 92 (58%) | 84 (35%) |
| Baseline dysphagia (grade 1 or 2) | | | 7 (5%) | 1(20%) | 8 (5%) | 84 (35%) |
| **Treatment variables** | | | | | | |
| Received combination proton and photon | | |  |  |  | 2 (1%) |
|  | Yes | | 130 (77%) | 5 (7%) | 135 (56%) |  |
|  | No | | 39 (23%) | 66 (93%) | 105 (44%) |  |
| Chemotherapy sequence | | |  |  |  | 1 (0%) |
|  | | Concurrent | 104 (61%) | 43 (61%) | 147 (61%) |  |
|  | | Sequential | 40 (23%) | 22 (31%) | 62 (26%) |  |
|  | | None | 27 (16%) | 5 (7%) | 32 (13%) |  |
| Surgery (yes) | | | 8 (5%) | 3 (4%) | 11 (5%) | 1 (0%) |
| Durvalumab (yes)* | | | 56 (67%) | 18 (60%) | 74 (66%) | 45 (29%) |
| Mean radiation dose received (Gy) | | | 58.4 (5.4) | 57.3 (6.0) | 58.1 (5.6) | 2 (1%) |
| Fractionation daily frequency | | |  |  |  | 0 (0%) |
|  | | Once daily | 157 (92%) | 60 (85%) | 217 (90%) |  |
|  | | Twice daily | 14 (8%) | 11 (16%) | 25 (10%) |  |
| Percentages may not total 100% due to rounding.  *Percentage of patients with stage III NSCLC that received durvalumab.  Abbreviations: NSCLC = non-small cell lung cancer, SCLC = small cell lung cancer, SD = standard deviation, WHO-PS = World Health Organisation Performance Status. | | | | | | |

**Appendix B: Covariate balance results for matching and propensity score weighting**

| **Variable** | **Proton (Mean)** | | **Photon (Mean)** | | **Standardised Mean difference** | | **Variance ratio** | | **T-test p-value** | | **KS statistic** | | **KS bootstrap p-value** | |
| --- | --- | --- | --- | --- | --- | --- | --- | --- | --- | --- | --- | --- | --- | --- |
|  | **Before** | **After** | **Before** | **After** | **Before** | **After** | **Before** | **After** | **Before** | **After** | **Before** | **After** | **Before** | **After** |
| **Genetic Matching** | | | | | | | | | | | | | | |
| Age at start of radiotherapy | 67.567 | 67.567 | 67.028 | 67.386 | 0.058 | 0.019 | 1.263 | 1.742 | 0.658 | 0.700 | 0.091 | 0.093 | 0.634 | 0.330 |
| Stage III/IV (vs stage I/II) | 0.848 | 0.848 | 0.958 | 0.848 | -0.305 | 0.000 | 3.160 | 1.000 | 0.003 | 1.000 | 0.110 | 0.000 | N/A | N/A |
| WHO PS 2+ (vs 0/1) | 0.175 | 0.175 | 0.239 | 0.181 | -0.168 | -0.015 | 0.788 | 0.975 | 0.278 | 0.564 | N/A | N/A | N/A | N/A |
| Baseline dyspnoea grade I/II (vs none) | 0.556 | 0.556 | 0.577 | 0.561 | -0.044 | -0.012 | 1.004 | 1.003 | 0.756 | 0.858 | N/A | N/A | N/A | N/A |
| Baseline EQ-5D-5L | 0.798 | 0.798 | 0.816 | 0.799 | -0.111 | -0.005 | 1.511 | 1.412 | 0.370 | 0.893 | 0.095 | 0.099 | 0.578 | 0.258 |
| **Propensity score weighting** | | | | | | | | | | | | | | |
| Age at start of radiotherapy | 67.567 | 67.439 | 67.028 | 67.065 | 0.060 | 0.041 | 1.262 | 1.151 | 0.657 | 0.766 | 0.091 | 0.060 | 0.806 | 0.995 |
| Stage III/IV (vs stage I/II) | 0.848 | 0.869 | 0.958 | 0.943 | -0.338 | -0.226 | 3.190 | 2.092 | 0.018 | 0.150 | 0.110 | 0.073 | 0.018 | 0.150 |
| WHO PS 2+ (vs 0/1) | 0.175 | 0.181 | 0.239 | 0.229 | -0.162 | -0.121 | 0.792 | 0.840 | 0.254 | 0.406 | 0.064 | 0.048 | 0.254 | 0.406 |
| Baseline dyspnoea grade I/II (vs none) | 0.556 | 0.549 | 0.577 | 0.561 | -0.044 | -0.024 | 1.012 | 1.008 | 0.755 | 0.870 | 0.022 | 0.012 | 0.755 | 0.870 |
| Baseline EQ-5D-5L | 0.798 | 0.802 | 0.816 | 0.811 | -0.117 | -0.062 | 1.510 | 1.294 | 0.367 | 0.655 | 0.095 | 0.070 | 0.750 | 0.973 |
